# Development of a Rapid Isothermal Assay for Detection of Adenovirus Types Important in Respiratory Infections

**DOI:** 10.1111/irv.70142

**Published:** 2025-08-02

**Authors:** Benedikt Beilstein, Iris Bachmann, Martin Spiegel, Frank T. Hufert, Gregory Dame

**Affiliations:** ^1^ Institute of Microbiology and Virology Brandenburg Medical School Theodor Fontane (MHB) Senftenberg Germany; ^2^ Infection Biology Unit German Primate Center Göttingen Germany; ^3^ Brandenburg University of Technology Cottbus‐Senftenberg (BTU) Senftenberg Germany; ^4^ Faculty of Health Sciences, Joint Faculty of BTU Cottbus‐Senftenberg MHB Theodor Fontane and University of Potsdam Potsdam Germany; ^5^ Institute for Laboratory Medicine OGD Neuruppin Neuruppin Germany

**Keywords:** HAdV‐B, HAdV‐C, HAdV‐E, human adenovirus (HAdV), penton gene, recombinase polymerase amplification (RPA), respiratory infections

## Abstract

**Background:**

Nucleic acid amplification tests (NAATs) for human adenoviruses (HAdVs) causing respiratory infections usually target the hexon gene. However, new HAdV types with substantial variations in the hexon gene may not be detected. Thus, we focus on NAATs based on a conserved region in the penton gene to detect all HAdV types causing respiratory infections.

**Methods:**

A highly conserved region at the 3′ end of the penton gene was chosen as a target for NAAT. Primers and probes for quantitative polymerase chain reaction (qPCR) and isothermal recombinase polymerase amplification (RPA) were designed for the detection of all HAdV types causing respiratory infections.

**Results:**

Two highly sensitive qPCR assays were established, one for the detection of HAdV‐E4 and HAdV‐B types and another for the detection of HAdV‐C types (LOD < 10 standard DNA copies for both assays). Furthermore, a one‐tube RPA with a universal RPA probe was developed for rapid detection of all HAdV types causing respiratory infections (LOD ≤ 244 standard DNA copies). All three assays were used for testing clinical nasopharyngeal swabs obtained from SARS‐CoV‐2‐negative children with respiratory disease symptoms. Eight out of 243 samples tested were found to be HAdV positive by qPCR and by one‐tube RPA, except for one sample with a very low viral load of 30 genome equivalents.

**Conclusions:**

Penton gene‐based NAAT systems were developed and successfully used for the detection of HAdV in clinical samples. The newly developed one‐tube RPA assay offers the possibility for rapid and simple detection of respiratory HAdV infections at the point of need.

**Abbreviations**
ARDS: acute respiratory distress syndrome
ARI: acute respiratory infection
CDC: Centers for Disease Control and Prevention
*C*_q_
: quantification cycle
HAdV: human adenovirus
HAdV‐BX: human adenovirus, Species B, Type X
HAdV‐CX: human adenovirus, Species C, Type X
HAdV‐E4: human adenovirus, Species E, Type 4
hMPV: human metapneumovirus
hRP: human RNase P
IBV: avian infectious bronchitis virus
LAMP: loop‐mediated isothermal amplification
LOD: lower limit of detection
MERS: Middle East respiratory syndrome coronavirus
NAAT: nucleic acid amplification test
NPA: nasopharyngeal aspirate
NTC: nontemplate control
POCT: point‐of‐care testing
(q)PCR: (quantitative) polymerase chain reaction
RAA: recombinase‐aided amplification
RPA: recombinase polymerase amplification
RSV: respiratory syncytial virus
RT: reverse transcription
SARS‐CoV‐2: severe acute respiratory syndrome coronavirus type 2
TT: threshold time

## 1 Introduction

Human adenoviruses (HAdVs) are responsible for 15% of acute respiratory infections (ARIs) in children requiring hospitalization and are the cause of 5%–10% (pediatric) and 1%–7% (adult) of all respiratory infections worldwide [1, 2, 3, 4]. Although HAdV infections are usually mild, they can have far‐reaching consequences, being one of the major causes of febrile seizures in children [5]. Especially in immunocompromised patients, in 10%–30% of cases, a disseminated infection leads to severe respiratory failure. Here, the mortality rate of severe HAdV pneumonia can rise over 50% [4]. Likewise, the mortality rate of HAdV infection in stem cell transplant patients is approximately 26%, whereas in cases with pneumonia, the mortality rate rises to 50% [6]. Furthermore, in children diagnosed with pneumonia and requiring intensive care support, 10% of the cases are caused by HAdV [7]. Additionally, respiratory HAdV infections in immunocompromised patients can lead to severe secondary complications, like hepatitis, gastroenteritis, and hemorrhagic cystitis [8, 9]. HAdVs are nonenveloped DNA viruses that remain infectious for long periods on environmental surfaces and that are resistant to many disinfectants [10]. Outbreaks of HAdV pneumonia due to insufficient hygiene management have been described in military [11] and long‐term care settings [12] and in intensive care units [13, 14].

HAdVs can be phylogenetically classified into seven species (A–G) with over 100 different types. Epidemiological studies have identified types of Species B (*Mastadenovirus blackbeardi*), C (*Mastadenovirus caesari*), and E (*Mastadenovirus exoticum*) as the HAdVs causing respiratory infections, with HAdV‐2 (Species C), HAdV‐4 (Species E), and HAdV‐3, HAdV‐7, and HAdV‐14 (Species B) being the most important ones [1, 15, 16]. Direct sequencing of different clinical samples (e.g., nasopharyngeal aspirate (NPA), blood, and stool) revealed that only HAdV Species B, C, and E, but no other HAdV species, are present in clinical NPA [17]. The HAdV genome, which is approximately 35 kb in size, encodes a large number of proteins, including those that form the icosahedral capsid [18]. The icosahedral capsid is mainly composed of 240 hexon protein trimers forming the facets, whereas at the vertices, 12 penton base protein pentamers are located, which are associated with 12 fiber trimers [19]. This complex forms a structure with species‐specific antigens for viral attachment to cellular receptors.

Currently, nucleic acid‐based HAdV detection systems mainly use the hexon protein gene as a detection target both in PCR [20, 21, 22, 23, 24, 25, 26] and in isothermal amplification techniques such as RPA and LAMP [26, 27, 28], which are especially suitable for point‐of‐care testing (POCT). However, over the last 15 years, a growing number of new HAdV types with considerable variations in the hexon gene have been identified [29, 30]. Therefore, hexon gene‐based pan‐HAdV assays may not reliably detect newer types associated with respiratory infections [31]. Penton gene–based assays could be a superior alternative because the penton gene possesses a highly conserved gene region across the different types [32, 33, 34] and may therefore allow the development of a single tube assay for the detection of all HAdV types relevant in respiratory infections.

In this study, we present novel nucleic acid amplification assays based on the penton gene for the detection of HAdV types of Species B, C, and E causing respiratory infections. Using a highly conserved region in the penton gene as a target for primer and probe binding, we developed qPCR assays for the detection of HAdV Species B and E types and a separate assay for HAdV Species C types. Furthermore, a qualitative isothermal single‐tube RPA assay was developed for the rapid detection of HAdV types of Species B, E, and C associated with respiratory infections. This assay is especially suitable for POCT due to a short turnaround time of 20 min (excluding sample preparation) and much simpler instrumentation compared to PCR. Finally, the newly developed RPA assay was successfully employed in testing clinical swab samples from SARS‐CoV‐2‐negative children with respiratory symptoms.

## 2 Methods

### 2.1 Primer and Probe Design for qPCR and RPA

Multiple sequence alignments of full‐length genomes from HAdVs causing respiratory infections (B3, 7, 11, 14, 16, 21, 34, 35, 55, 66, 68, C1, 2, 5, 6, 57, 89, and E4) were performed with Clustal Omega [35] to identify conserved regions.

Primers and probes for qPCR were designed using *Primer3* [36, 37], whereas primers and probes for RPA were designed using *PrimedRPA* [38, 39]. Primer–probe combinations were checked for secondary structure formation by *multiple primer analyzer* (Thermo Fisher Scientific, Waltham, MA, USA) and for specific binding to the HAdV target sequences by *NCBI BLAST* [40]. All primers and probes were purchased from biomers.net (Ulm, Germany) (Table S2).

### 2.2 Adenovirus Penton Gene DNA Standards

The penton DNA of HAdV‐B7 (GenBank No.: JX423383), HAdV‐B14 (GenBank No.: MK568772), HAdV‐E4 (2) (GenBank No.: KY996450), and HAdV‐C2 (GenBank No.: JX173084) were synthesized and inserted into plasmids (Thermo Fisher GeneArt, Regensburg, Germany). Each DNA insert was sequenced to verify the integrity of the penton gene sequences (Microsynth Seqlab, Göttingen, Germany). DNA fragments containing the penton genes were obtained by *Sfi*I (Thermo Fisher Scientific) digest and isolated using the Zymoclean Gel DNA Recovery Kit (Zymo Research Europe, Freiburg, Germany). The purified DNA was quantified using the PicoGreen dsDNA assay (Thermo Fisher Scientific). For HAdV‐B16 (GenBank No.: JN860680) and HAdV‐B68 (GenBank No.: AY601636), a 307‐bp fragment of the penton gene with 100% sequence identity between both types containing the PCR and RPA amplicons was synthesized (Thermo Fisher GeneArt) and used as a standard.

### 2.3 RT‐qPCR, qPCR, and RPA Assays

For human RNase P (hRP) mRNA detection [41], the Luna one‐step RT‐qPCR kit (E3006, NEB) was used according to the manufacturer's protocol with 1‐μL sample, 200‐nM probe, and 400 nM of each primer. Likewise, for HAdV qPCR assays, Luna Universal Probe qPCR Master Mix (M3004, NEB, Ipswich, MA, USA) was used with 1‐μL sample, 200‐nM probe, and 400 nM of each primer. PCR‐grade water (Carl Roth, Karlsruhe, Germany) was used as the nontemplate control (NTC). The (RT‐)qPCR assays were performed in a LightCycler 480 II (Roche Diagnostics, Mannheim, Germany) as follows: PCR: initial denaturation at 95°C for 60 s followed by two‐step amplification for 45 cycles, each cycle consists of denaturation at 95°C for 15 s and extension at 60°C for 30 s with fluorescence measurement; RT‐PCR: reverse transcription step at 55°C for 10 min followed by the same temperature protocol as described for PCR. Quantification cycle threshold (*C*_q_) values were determined using the integrated second derivative maximum method of the LightCycler 480 software (Roche Diagnostics).

For HAdV RPA assays, RPA was carried out using the TwistAmp exo kit (TwistDx, Maidenhead, UK) in a final volume of 50 μL with a 2‐μL sample according to the manufacturer's protocol. Primer and probe concentrations are listed in Table 1. RPA reactions were performed at 40°C for 20 min in an ESEQuant TS 1 fluorometer (Qiagen Dialunox, Stockach, Germany) with an additional mixing step 90 s after reaction start. PCR‐grade water (Carl Roth) was used as NTC. RPA raw fluorescence data were normalized to correct for background noise by subtracting the first fluorescence value after mixing from all raw fluorescence values. RPA threshold was set based on the data from the NTC measurements and calculated according to Frey et al. [42]. The time at which the fluorescence curve intersected the threshold line was defined as threshold time (TT).

**TABLE 1:** Primer and probe concentrations that were found to be optimal for assay performance in the indicated RPA assays.

RPA assay	Probe concentration (nM)	HAdV‐B and HAdV‐E primer concentration (nM)		HAdV‐C primer concentration (nM)	
		Forward	Reverse	Forward	Reverse
HAdV‐B + E RPA	120	420	840	—	—
HAdV‐C RPA	120	—	—	420	630
One‐tube RPA (HAdV‐B + C + E)	120	157.5	315	210	315

### 2.4 Analytical Sensitivity of qPCR and RPA

The analytical sensitivity of the qPCR and RPA assays was determined using decadic dilution series of each HAdV DNA standard (HAdV‐B7, B14, B16/68, E4, and C2), ranging from 10^7^ to 10^0^ standard DNA copies/μL. For the one‐tube RPA system, a dilution series comprising 10^4^–10^0^ standard DNA copies/μL was used. PCR assays were calibrated by linear regression analysis of *C*_q_ values plotted against the decimal logarithm of the respective standard DNA copies. Probit analysis [43] was used to determine the limit of detection (LOD) with 95% probability for each qPCR and RPA assay. All calculations were done in *R* using the qPCR package as described elsewhere [44] with eight replicates (qPCR) or seven replicates (RPA) for each standard DNA concentration and experiment.

### 2.5 Specificity of One‐Tube RPA

Genomic nucleic acid samples of 28 respiratory RNA and DNA viruses (Table 2) were used to determine the specificity of the one‐tube RPA. The viral genomes were either already available as nucleic acid extracts or were extracted using the QIAamp Viral RNA Mini Kit (Qiagen, Hilden, Germany). For the detection of viral RNA, 500 units of RevertAid reverse transcriptase (Thermo Fisher Scientific) were added to each RPA reaction mix. Testing of each viral RNA or DNA was performed in duplicate.

**TABLE 2:** Virus panel for specificity testing. Genomic RNA or DNA of 28 respiratory viruses was used for specificity testing in newly established RPA assays.

Virus name (virus type)	Type of genome
Influenza A virus^a^ (H1N1, H7N7, H5N1, H3N2, and H7N7)	RNA
Influenza B virus^a^ (Yamagata and Victoria)	RNA
Rhinovirus (Types C^a^, B5^a^, and A1^b^)	RNA
RSV (Type A^a^)	RNA
IBV M41^a^	RNA
hMPV^a^ (Types B2 and A1)	RNA
Parainfluenzavirus^a^ (Types 2, 10.3, and 4a)	RNA
SARS‐CoV‐2^b^	RNA
MERS‐CoV^a^	RNA
Adenovirus (Types C1^a^, E4^a^, A31^c^, B7^c^, B14^c^, C2^c^, D8^c^, D37^c^, and F41^c^)	DNA

Abbreviations: IBV: avian infectious bronchitis virus, hMPV: human metapneumonvirus, MERS: Middle East respiratory syndrome coronavirus, RSV: respiratory syncytial virus, SARS‐CoV‐2: severe acute respiratory syndrome coronavirus type 2.

^a^ Friedrich Loeffler Institute, Germany, and Quality Control for Molecular Diagnostics (QCMD, UK).

^b^ NATtrol Respiratory Verification Panel 2.1 (Zepto Metrix, USA).

^c^ German reference laboratory for adenoviruses, Hannover Medical School, Germany.

### 2.6 Clinical Samples

Leftover material (500 μL) of nasopharyngeal swab samples resuspended in 750‐μL BD Molecular Respiratory Sample Buffer (Becton Dickinson, Sparks, Maryland, USA) from children with respiratory disease symptoms and already tested negative for SARS‐CoV‐2 (Institute for Laboratory Medicine, OGD Neuruppin, Germany) was mixed with 500‐μL cobas omni Lysis Reagent (Roche Diagnostics, Mannheim, Germany) for storage at 4°C. Nucleic acids were extracted using QIAamp Viral RNA Mini Kit (Qiagen) according to the manufacturer's instructions. For the elution of purified nucleic acids, 30‐μL RNase‐free water was used. RT‐qPCR for the detection of hRP was performed as a control for the integrity and quality of the clinical samples [41]. Only hRP‐positive samples (*C*_q_ < 35) were tested for HAdV.

## 3 Results

### 3.1 HAdV Penton Amplicon Design for qPCR and RPA

**FIGURE 1:**
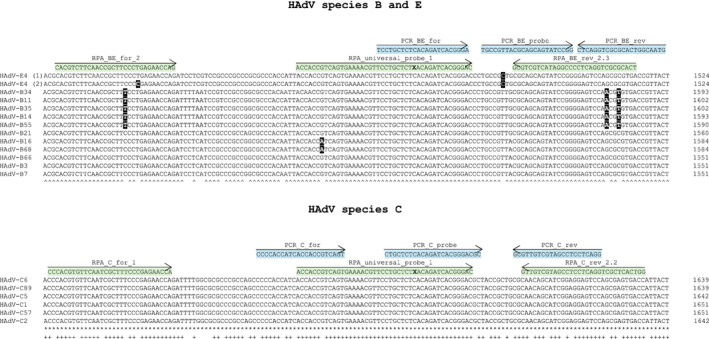
Primers and probes for the detection of human adenovirus (HAdV) types causing respiratory infections. A conserved region in the penton gene of HAdV types relevant in respiratory infections (Table S1) of Species B and E and Species C was identified by multiple alignment using Clustal Omega. Shown are representative sequences for each type. Sequences of HAdV‐E4 (2), HAdV‐B7, HAdV‐B14, HAdV‐B16, HAdV‐B68, and HAdV‐C2 correspond to sequences in GenBank accessions KY996450, JX423383, MK568772, JN860680, AY601636, and JX173084, which were also used for preparation of DNA standards. Sequence mismatches of primers and probes found in all penton gene sequences available in the nucleotide database of NCBI GenBank for each depicted HAdV type are shown in black squares. ^: identical nucleotides across types of Species B and Type E4, *: identical nucleotides across types of Species C, +: identical nucleotides across types of species B, Type E4 and types of Species C. Primers and probes (qPCR, depicted in blue; recombinase polymerase amplification [RPA], depicted in green, Table S2) were chosen with the help of Primer 3 and *PrimedRPA* software. X: abasic site for *Exonuclease* III cleavage in RPA probe.

Full genome alignments of HAdV types causing respiratory infections were performed to identify highly conserved regions. Three genes showing the highest homology within and across the HAdV species were identified: the DNA polymerase gene, the hexon gene, and the penton gene. The DNA polymerase gene was excluded from the amplicon design because the aligned sequences exhibit mismatches across the entire gene length, despite an overall high homology. Therefore, it was not possible to find a suitable amplicon region for RPA, which must be at least 100–120 bp in length [45]. Another multiple sequence alignment (Figure S1) of HAdV types associated with respiratory infections (11 types of species HAdV‐B, 6 types of species HAdV‐C, and 1 type of species HAdV‐E; Table S1) identified a highly homologous region in the penton gene. For each HAdV type, all penton gene sequences available in the nucleotide database of NCBI GenBank were aligned to identify possible sequence variations. The highest degree of homology was found for types within HAdV Species C, which exhibited 100% identity in the region of interest of the penton gene (Figure 1). Sequence identity across HAdV‐E4 and HAdV‐B types was 91.7%, whereas sequence identity across all relevant types of HAdV Species B, C, and E was 82.1% in the region of interest (Figure 1). Based on this result, amplicon regions in the penton gene were chosen for the detection of HAdV Types B, C, and E in qPCR and RPA (Figure 1).

For qPCR, two systems were developed. The first system uses a primer pair and a probe for the detection of HAdV‐E4 and HAdV‐B types relevant in respiratory infections. The second system was designed for optimal detection of HAdV‐C types relevant in respiratory infections and uses a different primer pair and a separate probe (Figure 1).

For RPA, different primer–probe combinations were tested (data not shown). We were able to identify a universal RPA probe, which can be used for the detection of all HAdV types (types of Species B and C and Type E4) causing respiratory infections. The universal RPA probe was combined either with a primer pair for the detection of HAdV‐E4 and HAdV‐B types (B + E) or with another primer pair for the detection of HAdV‐C types (C) (Figure 1). Because the same probe was used in both assays, we also set out to combine both primer pairs and the probe in a one‐tube RPA assay (B + E + C) for the detection of HAdV‐E4, HAdV‐B types, and HAdV‐C types.

### 3.2 Analytical Sensitivity of qPCR Assays

**FIGURE 2:**
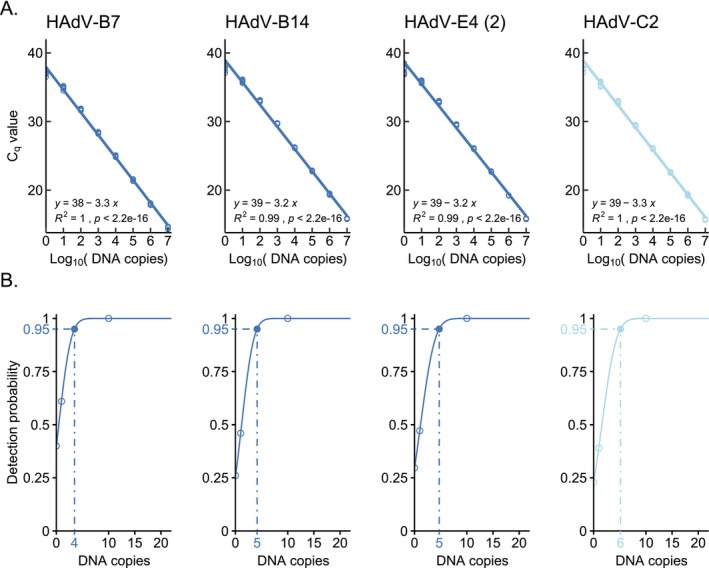
Analytical sensitivity of the qPCR assays. (A) Calibration curves calculated by linear regression for qPCR results using the penton gene standard DNA as template. HAdV‐qPCR results exhibit a linear dependency of *C*_q_ values with respect to Log_10_ (DNA copies) over the tested DNA standard range (10^7^–10^0^ standard DNA copies, *n* = 8 for each amount of standard DNA). (B) Probit analyses for the calculation of limits of detection (LODs). The LODs with 95% detection probability (0.95) are marked with dashed lines. Penton gene DNA standards tested: HAdV‐B7 (one mismatch in PCR_BE_rev primer), HAdV‐B14 (two mismatches in PCR_BE_rev primer), HAdV‐E4 (2) (one mismatch in PCR_BE_probe), and HAdV‐C2 (no mismatches in PCR primers and probe). Dark blue: detection by HAdV‐B + E qPCR. Light blue: detection by HAdV‐C PCR.

Linear regression analysis revealed that the newly developed HAdV PCR assays exhibited a reliable performance over the whole concentration range tested (10^7^–10^0^ standard DNA copies/μL; Figure 2). Using probit analysis, the LODs of the qPCR system for the detection of HAdV‐E4 and HAdV‐B types were determined to be four standard DNA copies (HAdV‐B7 standard), five standard DNA copies (HAdV‐B14 standard), and five standard DNA copies (HAdV‐E4 standard), whereas the LOD for the detection of HAdV‐C types in the HAdV‐C qPCR assay was determined to be six standard DNA copies (HAdV‐C2 standard) (Figure 2).

### 3.3 Limit of Detection of RPA Assays

First, a series of optimization experiments was performed for the RPA assays detecting HAdV‐E4 and HAdV‐B types (B + E) and HAdV‐C types (C), as well as for the one‐tube RPA assay (B + E + C) to determine the primer and probe concentrations that resulted in the highest possible fluorescence signals (Table 1).

By probit analysis, the LODs of the optimized HAdV‐B + E RPA system and the HAdV‐C RPA were determined (Figure S2). For the former, LODs of 25 standard DNA copies for HAdV‐B7, 37 standard DNA copies for HAdV‐B14, and 14 standard DNA copies for HAdV‐B16/68 were calculated, whereas the LOD for the HAdV‐E4 standard was determined to be 38 DNA copies. The optimized RPA system for HAdV‐C types exhibited an LOD of 239 standard DNA copies (HAdV‐C2 standard, Figure S2).

**FIGURE 3:**
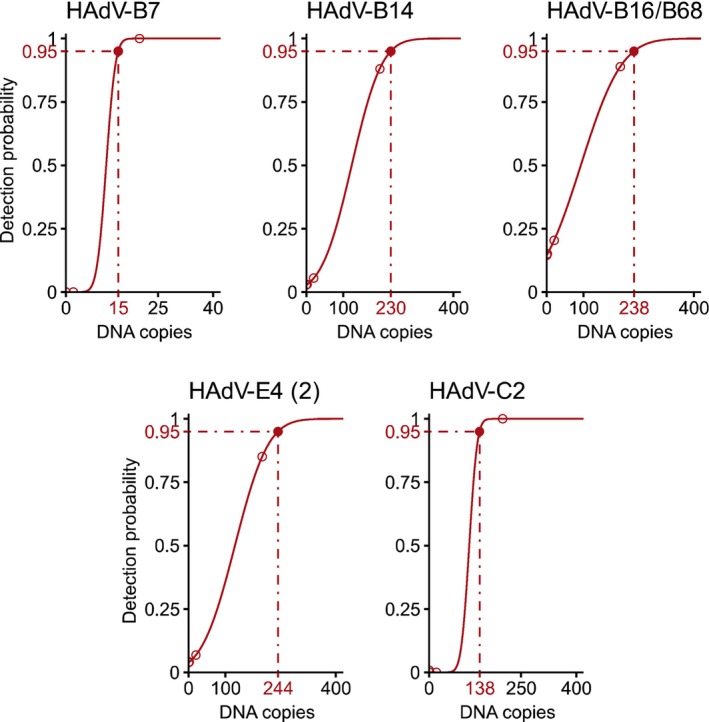
Limits of detection (LODs) of the one‐tube RPA assay. Penton gene DNA standards tested: HAdV‐B7 (no mismatches in RPA BE primers and RPA universal probe), HAdV‐B14 (one mismatch in RPA_BE_for_2 primer, two mismatches in RPA_BE_rev_2.3 primer), HAdV‐B16/86 (one mismatch in RPA_universal_probe_1), HAdV‐E4 (2) (one mismatch in RPA_BE_for_2 primer), and HAdV‐C2 (no mismatches in RPA C primers and RPA universal probe). Tested standard DNA range: 10^4^–10^0^ standard DNA copies, *n* = 7 for each amount of standard DNA. The LODs with 95% detection probability (0.95) calculated by probit analysis are marked with dashed lines.

For the one‐tube RPA, reaction temperature and time point of the additional mixing step were also optimized. Optimal results were achieved with a reaction temperature of 42°C (instead of 40°C) and additional mixing at 190 s (instead of 90 s) after reaction start. The optimized one‐tube RPA exhibited LODs of 15 standard DNA copies (HAdV‐B7), 230 standard DNA copies (HAdV‐B14), and 238 standard DNA copies (HAdV‐B16/68) for the detection of the HAdV‐B types (Figure 3), an LOD of 244 copies for the HAdV‐E4 DNA standard, and an LOD of 138 copies for the HAdV‐C2 DNA standard (Figure 3).

### 3.4 Specificity of the One‐Tube RPA Assay

The specificity of the one‐tube RPA was tested using a panel of genomic RNAs and DNAs of respiratory viruses and adenoviruses causing keratoconjunctivitis (*Mastadenovirus dominans* HAdV‐D8 and HAdV‐D37) or gastroenteritis (*Mastadenovirus faecalis* HAdV‐F41 and *Mastadenovirus adami* HAdV‐A31) (Table 2). For this purpose, RPA was performed with an additional RT step (RT‐RPA) [46, 47]. All respiratory RNA virus samples were negative in RT‐RPA when using the HAdV‐specific RPA primers and probe. Positive results were obtained for the respiratory adenovirus types HAdV‐B7, HAdV‐B14, HAdV‐C1, HAdV‐C2, and HAdV‐E4. Additionally, the one‐tube RPA detected the nonrespiratory adenovirus types HAdV‐A31, HAdV‐D8, and HAdV‐D37, whereas HAdV‐F41 remained negative.

### 3.5 Testing of Clinical Samples

Genomic DNA was extracted from 243 nasopharyngeal swab samples taken from children with respiratory disease symptoms but tested negative for SARS‐CoV‐2. First, the quality and integrity of the extracted nucleic acids were confirmed by a positive hRP RT‐PCR result, which was met by 81% (197/243) of the samples. These samples were tested using the newly established PCR and RPA assays. Eight out of 197 samples were HAdV‐C positive in qPCR. No sample was found to be positive for HAdV‐B or HAdV‐E4. The viral load of the positive samples was in the range of 1.3 × 10^1^ to 1.1 × 10^3^ genome equivalents/μL (Table 3). Seven of these HAdV‐positive samples were confirmed by one‐tube RPA. The sample with the lowest viral load of 1.3 × 10^1^ genome equivalents/μL according to the HAdV‐C PCR result remained negative in the one‐tube RPA, probably because the amount of viral DNA present in 2‐μL eluate (6.0 × 10^1^ genome equivalents) used as template in the RPA assay was below the determined LOD of 138 for HAdV‐C in the one‐tube RPA (Figure 3).

**TABLE 3:** HAdV testing of clinical samples. DNA extracts from 243 nasopharyngeal swab samples, taken from children with respiratory symptoms, were screened for the presence of human RNaseP‐ (hRP‐) mRNA to check the quality of the sample material. Nucleic acid extracts from the 197 hRP‐positive samples were further tested by HAdV‐B + E and HAdV‐C qPCR. Eight samples were found to be positive for HAdV‐C and were also tested by one‐tube RPA. Viral loads were calculated from the obtained *C*_q_ values using the calibration curve for the HAdV‐C qPCR (Figure 2). The qPCR assays were performed with 1‐μL eluate as template in a total reaction volume of 20 μL, whereas 2‐μL eluate in a total reaction volume of 50 μL were used for RPA.

Sample no.	qPCR, est. HAdV viral load (genome equivalents/μL)		One‐tube RPA result
	Eluate	Sample	
84	7.6 × 10^2^	3.3 × 10^2^	**+**
91	7.1 × 10^2^	3.0 × 10^2^	**+**
103	3.0 × 10^1^	1.3 × 10^1^	**−**
153	8.6 × 10^1^	3.7 × 10^1^	**+**
170	1.9 × 10^3^	8.1 × 10^2^	**+**
197	2.6 × 10^3^	1.1 × 10^3^	**+**
202	1.8 × 10^3^	7.7 × 10^2^	**+**
221	1.8 × 10^2^	7.7 × 10^1^	**+**

Abbreviations: HAdV: human adenovirus, RPA: recombinase polymerase amplification, +: positive in one‐tube RPA, −: negative in one‐tube RPA.

## 4 Discussion

With 15% of all ARIs in children requiring hospitalization, HAdV types causing respiratory infections are important pathogens. In particular, the emergence of severe courses with ARDS or disseminated infections with high mortality highlights the pathogenic potential [1, 2, 3, 5, 18, 48, 49]. In immunocompromised patients, especially children, respiratory HAdV types, namely, B3, B7, B11, C1, C2, and C5, can also lead to severe secondary complications like hepatitis, gastroenteritis, and hemorrhagic cystitis with potentially fatal outcomes [8, 9]. Several studies have demonstrated that HAdV types from Species B, C, and E are relevant in respiratory infections [1, 16, 17]. Because no approved antiviral therapy for adenovirus infection exists, early detection of these HAdV types, especially during epidemic outbreaks, is of great importance, as this can help to limit the spread of viruses and thereby limit the number of infections. In case of community‐based outbreaks, but also in case of nosocomial HAdV infections (especially in intensive care units), methods suitable for POCT can improve rapid detection of HAdVs, thereby allowing the setup of an effective infection prevention management that limits further transmission. Therefore, such methods should be able to detect all HAdVs associated with respiratory infections. A number of RPA or RAA assays for the detection of HAdVs have been published already. However, to our knowledge, none of these assays specifically detect all respiratory HAdV types. Either they cover only a subset of these types (most commonly from Species B) [23, 50, 51, 52, 53] or they are designed to detect all human pathogenic adenoviruses [54]. Furthermore, some of these assays are not suitable for POCT, because they are performed in combination with a second method (vertical flow microarray, qPCR, or CRISPR/CAS) to increase the sensitivity and specificity of the assay [54, 55, 56]. Up to now, nucleic acid‐based HAdV detection systems mainly use the gene of the hexon protein as a target [20, 21, 22, 23, 24, 25, 26]. Although sequence variations in the hexon protein gene can be helpful to discriminate HAdV types using NAATs, they might prevent simultaneous detection of different HAdV types causing the same disease complex. We therefore chose a conserved region in the penton gene (Figure 1 and Figure S1) to establish sensitive PCR assays for the detection of HAdV types causing respiratory infections, as well as the one‐tube RPA assay as an isothermal alternative suitable for POCT that can rapidly detect all HAdV types from Species B, E, and C relevant in respiratory infections.

For the very sensitive and quantitative HAdV detection, two PCR assays were established. Our aim was to minimize possible mismatches of primers and probes concerning the target sequences in the penton gene. Therefore, we established one qPCR assay for the detection of the closely related HAdV‐B and HAdV‐E types and one assay for the detection of the phylogenetically more distant but very homologous HAdV‐C types [57], causing respiratory infections. Interestingly, up to two mismatches in primer or probe sequences had no apparent impact on the analytical sensitivity of the qPCR assays. For both qPCR assays, we observed a constant performance over the tested concentration range of 10^0^–10^7^ standard DNA copies/μL and very similar LODs independent of the synthetic DNA standard used (Figure 2). Thus, the qPCR assay detecting HAdV‐B and HAdV‐E types (≤ 2 mismatches, Figure 1) and the qPCR assay detecting HAdV‐C types (no mismatch, Figure 1) are equivalent to each other in terms of analytical sensitivity, exhibit very low LODs (Figure 2), and can be used for the quantitative detection of low viral loads in clinical samples. Indeed, when testing nasopharyngeal swab samples from children with respiratory disease symptoms with the newly established qPCR assays, we were able to detect viral loads in the range of 1.3 × 10^1^ to 1.1 × 10^3^ HAdV‐C genome equivalents/μL sample.

For the rapid detection of all HAdV types relevant in respiratory infections, we established a qualitative RPA assay suitable for POCT. To this aim, we combined a probe suitable for the detection of HAdV‐B, HAdV‐C, and HAdV‐E types with two primer pairs, one for HAdV‐B and HAdV‐E types and one for HAdV‐C types. In this setting, up to three mismatches were tolerated in total for primer and probe sequences to ensure the detection of all HAdV types relevant in respiratory infections (Figure 1). Depending on the synthetic DNA standard used (representing the different HAdV types), LODs in the range of 15 standard DNA copies to 244 standard DNA copies were observed (Figure 3). In most cases, the one‐tube RPA was one order of magnitude less sensitive than the qPCR assays. However, this sensitivity should be sufficient for POCT settings, because the primary goal here is the early detection of severe cases with higher viral loads, which will allow us to implement infection prevention management that will limit further HAdV spread in time. It has been demonstrated that the viral load in swab samples from patients with severe HAdV respiratory infection is at least 5.0 × 10^2^ genome equivalents/μL sample [58]. The nasopharyngeal swab samples from children with respiratory disease symptoms that we tested contained viral loads between 1.3 × 10^1^ and 1.1 × 10^3^ genome equivalents of HAdV‐C/μL (Table 3). Despite the fact that five of the eight positive samples contained viral loads less than 5.0 × 10^2^ genome equivalents/μL we were able to detect these low viral loads with the one‐tube RPA assay except for the sample with the lowest value of 1.3 × 10^1^ genome equivalents/μL (Table 3). For HAdV‐C types, the one‐tube RPA seems to perform equally well with synthetic DNA standards and with viral DNA extracted from clinical samples: The clinical sample #153 with a low viral load of 3.7 × 10^1^ genome equivalents/μL was successfully detected as HAdV‐C‐positive by one‐tube RPA, although the reaction mix contained only 1.7 × 10^2^ genome equivalents, which is near the LOD of 138 determined by using a synthetic DNA standard.

To some extent, the one‐tube RPA assay also detects nonrespiratory HAdV types; however, not all of them are present in nasopharyngeal swab samples, NPA, or bronchoalveolar lavage [17, 59, 60]. Therefore, HAdV‐A31, which causes gastroenteritis and diarrhea, will not lead to false‐positive results when testing respiratory samples by the one‐tube RPA assay [61]. Additionally, HAdV Types D8 and D37, which cause mainly keratoconjunctivitis, were also detected. Interestingly, these types sometimes cause respiratory disease, especially in neonates and young children [62, 63]. Thus, the detection of these types in respiratory samples by the one‐tube RPA might be helpful to identify unusual cases of HAdV‐D8‐ and HAdV‐D37‐induced respiratory disease.

With respect to future multiplex RPA assays that simultaneously detect respiratory RNA and DNA viruses, we also tested whether the one‐tube RPA nonspecifically detects respiratory RNA viruses (Table 2) in an RT‐RPA setting. After reverse transcription of genomic viral RNA, none of the tested RNA viruses were detected using HAdV‐specific primers and probe, indicating that DNA generated by reverse transcription from viral RNA does not lead to false‐positive results when used as a template in the one‐tube assay.

## 5 Conclusions

Rapid detection of severe respiratory HAdV infections requires methods that can detect all relevant HAdV types in one assay. To this aim, we developed an isothermal RPA assay that is suitable for POCT due to its simple instrumentation and short turnaround time. The assay is capable of detecting all HAdV‐B, HAdV‐C, and HAdV‐E types causing respiratory infections with a sensitivity that reliably captures viral loads that occur in severe respiratory HAdV infections. Furthermore, the one‐tube RPA assay is highly specific for adenoviruses, making it suitable for multiplex assays detecting different respiratory pathogens simultaneously.

## Author Contributions

**Benedikt Beilstein:** formal analysis, investigation, methodology, software, writing – original draft. **Iris Bachmann:** conceptualization, formal analysis, investigation, methodology, software, validation, visualization, writing – review and editing. **Martin Spiegel:** conceptualization, investigation, software, supervision, validation, visualization, writing – review and editing. **Frank T. Hufert:** funding acquisition, project administration, resources, writing – review and editing, supervision. **Gregory Dame:** conceptualization, funding acquisition, project administration, resources, supervision, writing – review and editing.

## Ethics Statement

All experiments were performed in compliance with relevant laws and institutional guidelines and in accordance with the ethical standards of the Declaration of Helsinki.

## Conflicts of Interest

The authors declare no conflicts of interest.

## Peer Review

The peer review history for this article is available at https://www.webofscience.com/api/gateway/wos/peer‐review/10.1111/irv.70142.

## Supporting information


**Table S1.** List of HAdVs causing respiratory infection. The following genomic sequences of HAdV types associated with respiratory infections were used for the selection of the penton target sequence and the primer–probe design for PCR and RPA.
**Figure S1.** Homology of HAdV penton and hexon genes. Multiple sequence alignments were performed with Clustal Omega for (1) HAdV Species B and Type E4; (2) HAdV Species C; and (3) HAdV Species B and C and Type E4 to identify a highly homologous region suitable as an amplicon for qPCR and RPA. The similarity of the compared sequences (complete list, Table S1) to the consensus sequence is shown, ranging from 0% to 100% (white to blue). The chosen amplicon regions within the penton gene region are highlighted in yellow for RPA and in red for PCR.
**Table S2.** Primers and probes for HAdV‐RPA and qPCR. Overview of the designed PCR and RPA primers and probes. For RPA, exo‐IQ probes 2 were designed, which are labeled with 6‐Fam at an internal deoxythymidine residue and with a quencher (BMN‐Q535) that is located between Positions 2 and 3 downstream of the fluorophore. In between fluorophore and quencher, an abasic site (X) for cleavage with Exonuclease III replaces the regular nucleotide. For qPCR, probes were designed that are labeled with 6‐Fam at the 5′ end and double quenched with BMN‐Q535 at the 3′ end and internally between nucleotide positions 8 and 9.
**Figure S2.** Limits of detection (LODs) of the individual HAdV‐RPA assays. (A) HAdV‐B + E RPA assay. Detection limits were determined with synthetic penton gene DNA standards HAdV‐B7 (no mismatches in RPA BE primers and RPA universal probe), HAdV‐B14 (one mismatch in RPA_BE_for_2 primer and two mismatches in RPA_BE_rev_2.3 primer), HAdV‐B16/86 (one mismatch in RPA_universal_probe_1), and HAdV‐E4 (2) (one mismatch in RPA_BE_for_2 primer). B. HAdV‐C RPA assay. The detection limit was determined with the HAdV‐C2 standard (no mismatches in RPA C primers and RPA_universal_probe). Tested standard DNA range: 10^4^–10^0^ standard DNA copies, *n* = 7 for each amount of standard DNA. The LODs with 95% detection probability (0.95) calculated by probit analysis are marked with dashed lines.

## Data Availability

All data generated and analyzed during this study are included in this article and its Supporting Information. Raw data are available from the authors upon request.
