# Supplementary material for: Development of a Rapid Isothermal Assay for Detection of Adenovirus Types Important in Respiratory Infections
Source: Influenza Other Respir Viruses. 2025 Aug 2;19(8):e70142. doi: 10.1111/irv.70142 (PMC12317340; doi:10.1111/irv.70142)
Supplement: Supplementary file 1 — Table S1. List of HAdVs causing respiratory infection. The following genomic sequences of HAdV types associated with respiratory infections were used for the selection of the penton target sequence and the primer–probe design for PCR and RPA. Figure S1. Homology of HAdV penton and hexon genes. Multiple sequence alignments were performed with Clustal Omega for (1) HAdV Species B and Type E4; (2) HAdV Species C; and (3) HAdV Species B and C and Type E4 to identify a highly homologous region suitable as an amplicon for qPCR and RPA. The similarity of the compared sequences (complete list, Table S1) to the consensus sequence is shown, ranging from 0% to 100% (white to blue). The chosen amplicon regions within the penton gene region are highlighted in yellow for RPA and in red for PCR. Table S2. Primers and probes for HAdV‐RPA and qPCR. Overview of the designed PCR and RPA primers and probes. For RPA, exo‐IQ probes 2 were designed, which are labeled with 6‐Fam at an internal deoxythymidine residue and with a quencher (BMN‐Q535) that is located between Positions 2 and 3 downstream of the fluorophore. In between fluorophore and quencher, an abasic site (X) for cleavage with Exonuclease III replaces the regular nucleotide. For qPCR, probes were designed that are labeled with 6‐Fam at the 5′ end and double quenched with BMN‐Q535 at the 3′ end and internally between nucleotide positions 8 and 9. Figure S2. Limits of detection (LODs) of the individual HAdV‐RPA assays. (A) HAdV‐B + E RPA assay. Detection limits were determined with synthetic penton gene DNA standards HAdV‐B7 (no mismatches in RPA BE primers and RPA universal probe), HAdV‐B14 (one mismatch in RPA_BE_for_2 primer and two mismatches in RPA_BE_rev_2.3 primer), HAdV‐B16/86 (one mismatch in RPA_universal_probe_1), and HAdV‐E4 (2) (one mismatch in RPA_BE_for_2 primer). B. HAdV‐C RPA assay. The detection limit was determined with the HAdV‐C2 standard (no mismatches in RPA C primers and RPA_universal_probe). Tested stan [file IRV-19-e70142-s001.docx]

SUPPORTING INFORMATION FOR THE ARTICLE

**Development of a rapid isothermal assay for detection of adenovirus types important in respiratory infections**

Benedikt Beilstein^1^ | Iris Bachmann^1^ | Martin Spiegel^1,2, *^ | Frank T. Hufert^1,3,4,5^ | Gregory Dame^1,3,4, *^

^1^Institute of Microbiology and Virology, Brandenburg Medical School Theodor Fontane (MHB), Universitätsplatz 1, 01968 Senftenberg, Germany | ^2^German Primate Center, Infection Biology Unit, Kellnerweg 4, 37077 Göttingen, Germany | ^3^Brandenburg University of Technology Cottbus–Senftenberg (BTU), Universitätsplatz 1, 01968 Senftenberg, Germany | ^4^ Faculty of Health Sciences, Joint Faculty of BTU Cottbus–Senftenberg, MHB Theodor Fontane and University of Potsdam, Karl-Liebknecht-Str. 24-25, 14476 Potsdam, Germany | ^5^ Institute for Laboratory Medicine, OGD Neuruppin, Fehrbelliner Str. 38, 16816 Neuruppin, Germany

**Correspondence** Martin Spiegel (martin.spiegel@mhb-fontane.de) | Gregory Dame (gregory.dame@mhb-fontane.de)

**Overview to supplement**

| **Content / specification** | **Supplementary Figure / Table** | **Page** |
| --- | --- | --- |
| List of HAdVs causing respiratory infection | Table S1 | 2 |
| Homology of HAdV penton and hexon genes | Figure S1 | 2 |
| Primers and probes for HAdV-RPA and qPCR | Table S2 | 3 |
| Detection limits of the individual HAdV-RPA assays | Figure S2 | 3 |

**References**

1. Heim A. Adenoviren. In: Suerbaum S, Burchard G-D, Kaufmann SHE, Schulz TF, eds. Medizinische Mikrobiologie und Infektiologie. 8th ed. Springer-Verlag GmbH; 2016:chap 70. doi:10.1007/978-3-662-48678-8_70

2. Behrmann O, Bachmann I, Spiegel M, et al. Rapid detection of SARS-CoV-2 by low volume real-time single tube reverse transcription recombinase polymerase amplification using an exo probe with an internally linked quencher (exo-IQ). *Clin Chem*. 2020;66(8):1047-54. doi:10.1093/clinchem/hvaa116

**SUPPLEMENTARY TABLE S1 | List of HAdVs causing respiratory infection.** The following genomic sequences of HAdV types associated with respiratory infections were used for the selection of the penton target sequence and the primer-probe design for PCR and RPA.

| **HAdV** | | **NCBI Accession No.** | **Strain specification** | **Gene region** | |
| --- | --- | --- | --- | --- | --- |
| **species** | **type** |  |  | **Hexon base** | **Penton base** |
| B | 3^a^ | NC_011203.1^b^ | Ad 3 prototype GB | 18418-21252 | 13905-15539 |
| B | 7^a^ | AC_000018.1^b^ | Isolate China, 2003 | 18666-21470 | 14153-15787 |
| B | 11^a^ | NC_011202.1^b^ | ATCC-VR12, strain Slobitski | 18254-21100 | 13682-15367 |
| B | 14^a^ | OR777166.1 | Isolate USA, 2010 | 18247-21084 | 13693-15369 |
| B | 16^a^ | JN860680.1 | Strain E26, EGY | 18342-21164 | 13797-15464 |
| B | 21^a^ | PP084098.1 | Isolate USA, 2012 | 18410-21262 | 13877-15520 |
| B | 34^a^ | OR777179.1 | Isolate USA, 2015 | 18238-21093 | 13679-15355 |
| B | 35^a^ | AC_000019.1^b^ | ATCC VR‑718, strain Holden | 18257-21115 | 13690-15375 |
| B | 55^a^ | MT806170.1 | Isolate CHN, 2019 | 18193-21018 | 13626-15299 |
| B | 66^a^ | JN860676.1 | Strain 87-922, ARS | 18396-21200 | 13882-15516 |
| B | 68^a^ | JN860678.1 | Strain Arg 827/04, ARS | 18457-21291 | 13909-15576 |
| C | 1^a^ | AC_000017.1^b^ | ATCC VR-1, strain Adenoid 71 | 18861-21755 | 14166-15890 |
| C | 2^a^ | NC_001405.1^b^ | HAdV 2 consensus sequence | 18838-21744 | 14151-15866 |
| C | 5^a^ | AC_000008.1^b^ | Strain AD. 75 | 18842-21700 | 14156-15871 |
| C | 6^a^ | OR753103.1 | Isolate USA, 2010 | 18844-21735 | 14159-15871 |
| C | 57^a^ | LC062716.1 | Strain Osaka, JPN | 18851-21730 | 14158-15882 |
| C | 89^a^ | OR777171.1 | Isolate USA, 2010 | 18782-21691 | 14101-15813 |
| E | 4^a^ | KY996450.1 | Isolate 12-12752 (NY7) USA, 2012 | 18177-20987 | 13758-15365 |
| E | 4^a^ | NC_003266.2^b^ | Vaccine strain CL 68578 | 18248-21058 | 13815-15422 |

^a^ most common HAdV types causing respiratory tract infections, according to Heim ^1^; ^b^ NCBI Reference sequence of the respective HAdV type


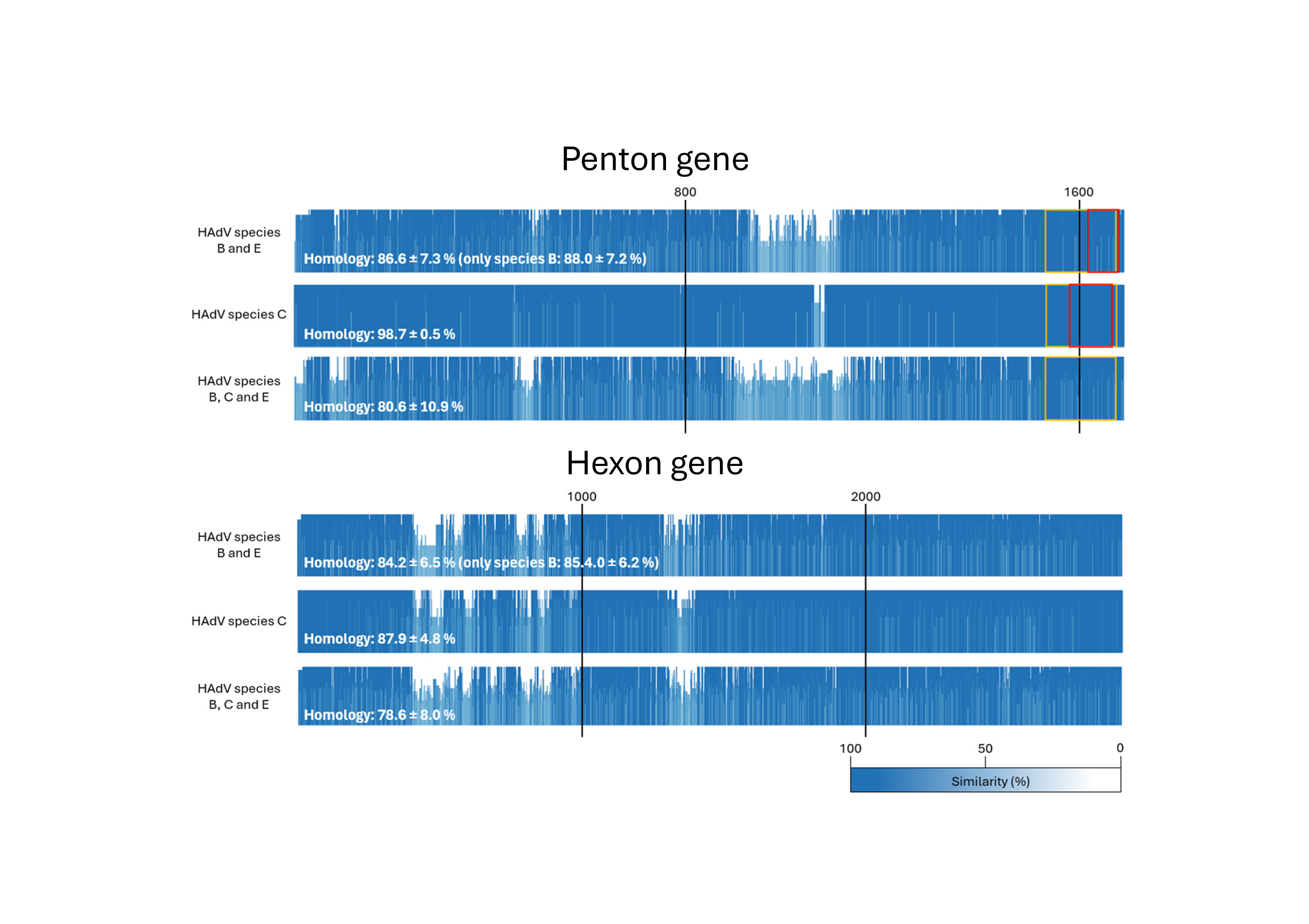


**SUPPLEMENTARY FIGURE S1 | Homology of HAdV penton and hexon genes.** Multiple sequence alignments were performed with Clustal Omega for 1) HAdV species B and type E4, 2) HAdV species C and 3) HAdV species B, C and type E4 to identify a highly homologous region suitable as amplicon for qPCR and RPA. The similarity of the compared sequences (complete list, Table S1) to the consensus sequence is shown, ranging from 0 – 100 % (white to blue). The chosen amplicon regions within the penton gene region are highlighted in yellow for RPA and in red for PCR.

**SUPPLEMENTARY TABLE S2 | Primers and probes for HAdV-RPA and qPCR**. Overview of the designed PCR and RPA primers and probes. For RPA, exo-IQ probes ^2^ were designed, which are labeled with 6-Fam at an internal deoxythymidine residue and with a quencher (BMN-Q535) that is located between position 2 and 3 downstream of the fluorophore. In between fluorophore and quencher an abasic site (X) for cleavage with *Exonuclease*III replaces the regular nucleotide. For qPCR, probes were designed that are labeled with 6-Fam at the 5’ end and double quenched with BMN-Q535 at the 3’ end and internally between nucleotide positions 8 and 9.

| **Primer-probe system** | **Oligonucleotide** | **Sequence 5’ → 3’** |
| --- | --- | --- |
| RPA set for species HAdV‑B and -E | RPA_BE_for_2 | CACGTCTTCAACCGCTTCCCTGAGAACCAG |
|  | RPA_BE_rev_2.3 | TCACGCGCTGGACTCCCCGGATACTGCTGCG |
| RPA set for species HAdV‑C | RPA_C_for_1 | CCCACGTGTTCAATCGCTTTCCCGAGAACC |
|  | RPA_C_rev_2.2 | GGTCACTCGCTGGACTCCTCCGATGCTGTTG |
| Universal RPA probe for species HAdV-B, -C and -E | RPA_universal_probe | ACCACCGTCAGTGAAAACGTTCCTGCTC (dT-Fam) X A (BMNQ535) CAGATCACGGGAC |
| PCR set for species HAdV‑B and -E | PCR_BE_for | TCCTGCTCTCACAGATCACGGGA |
|  | PCR_BE_rev | GTAACGGTCACGCGCTGGACTC |
|  | PCR_BE_probe | (6-Fam) TGCCGTTA (BMNQ535) CGCAGCAGTATCCGG (BMNQ535) |
| PCR set for species HAdV‑C | PCR_C_for | CCCCACCATCACCACCGTCAGT |
|  | PCR_C_rev | GGACTCCTCCGATGCTGTTGCG |
|  | PCR_C_probe | (6-Fam) CTGCTCTC (BMN-Q535) ACAGATCACGGGACGC (BMNQ535) |

for: forward primer; rev: reverse primer; dT: deoxythymidine; (6-)Fam: (6-Carboxy-)Fluorescein; X: abasic site (tetrahydrofuran) for *Exonuclease* III cleavage; BMN‑Q535: *‘biomers.net’* quencher (absorption maximum: 535 nm)


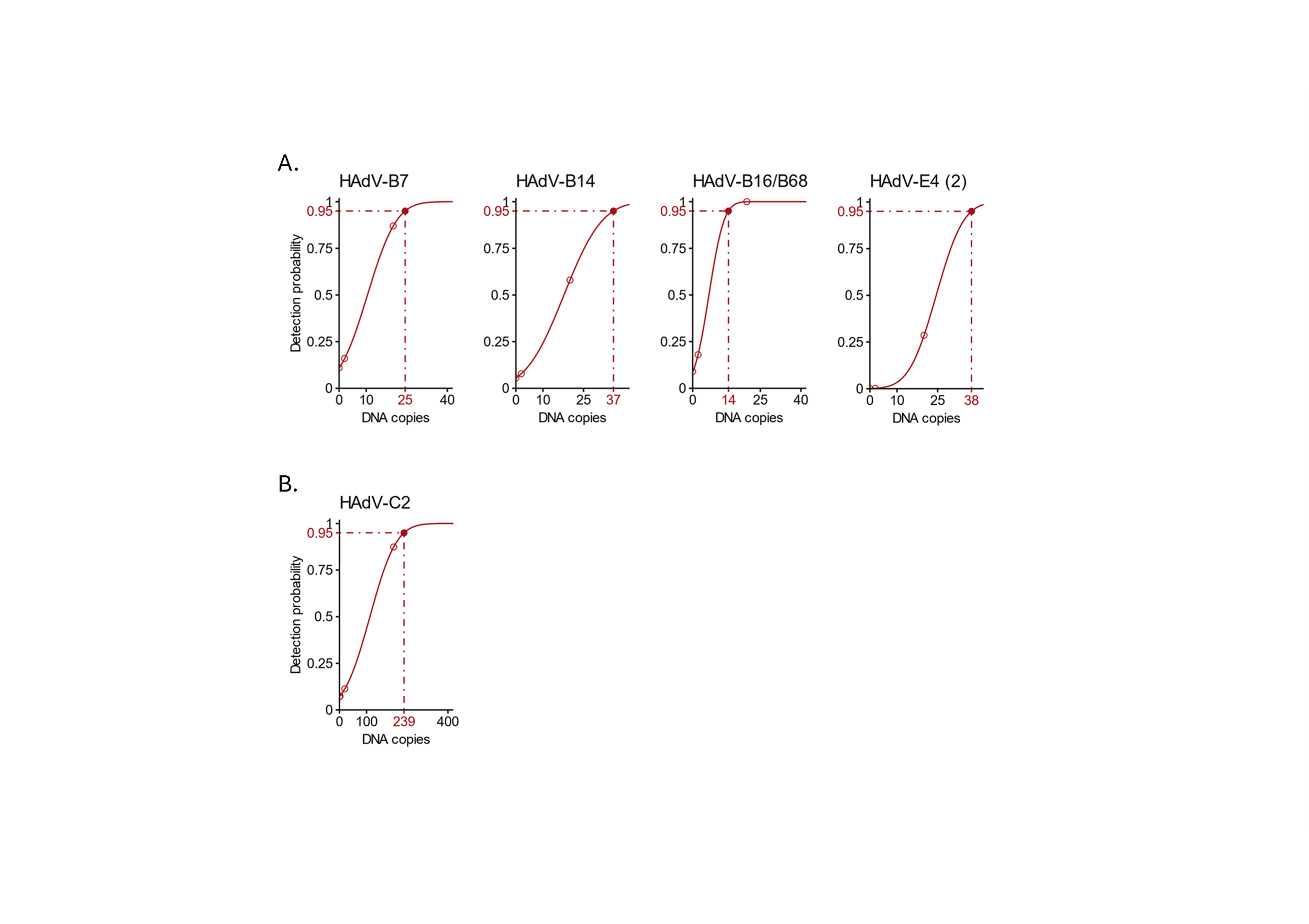


**SUPPLEMENTARY FIGURE S2 | Limits of detection (LODs) of the individual HAdV-RPA assays.** A. HAdV-B+E RPA assay. Detection limits were determined with synthetic penton gene DNA standards HAdV-B7 (no mismatches in RPA BE primers and RPA universal probe), HAdV-B14 (one mismatch in RPA_BE_for_2 primer, two mismatches in RPA_BE_rev_2.3 primer), HAdV-B16/86 (one mismatch in RPA_universal_probe_1), HAdV-E4 (2) (one mismatch in RPA_BE_for_2 primer). B. HAdV-C RPA assay. Detection limit was determined with the HAdV-C2 standard (no mismatches in RPA C primers and RPA_universal_probe). Tested standard DNA range: 10^4^ to 10^0^ standard DNA copies, n=7 for each amount of standard DNA. The LODs with 95% detection probability (0.95) calculated by probit analysis are marked with dashed lines.
